# Acute and subchronic exposure to urban atmospheric pollutants aggravate acute respiratory failure in infants

**DOI:** 10.1038/s41598-023-43670-1

**Published:** 2023-10-06

**Authors:** Amanda Barbosa Neto, Alexandre A. Ferraro, Sandra E. Vieira

**Affiliations:** Faculdade de Medicina, Universidade de São Paulo, São Paulo, Brazil

**Keywords:** Health care, Medical research

## Abstract

Urban air pollution is a major factor that affects the respiratory health of children and adolescents. Less studied is exposure during the first two years of life. This study analyzed the influence of acute and subchronic exposure to urban air pollutants on the severity of acute respiratory failure (ARF) in the first two years of life. This population-based study included 7364 infants hospitalized with ARF. Acute exposure was considered to have occurred 1, 3 and 7 days before hospitalization and subchronic exposure was considered the mean of the last 30 and 60 days. We found that for acute exposure, significant increases in days of hospitalization (LOS) occurred at lag 1 day for NO_2_ (0.24), SO_2_ (6.64), and CO (1.86); lag 3 days for PM_10_ (0.30), PM_2.5_ (0.37), SO_2_ (10.8), and CO (0.71); and lag 7 days for NO_2_ (0.16), SO_2_ (5.07) and CO (0.87). Increases in the risk of death occurred at lag 1 day for NO_2_ (1.06), SO_2_ (3.64), and CO (1.28); and lag 3 days for NO_2_ (1.04), SO_2_ (2.04), and CO (1.19). Subchronic exposures at 30 and 60 days occurred for SO_2_ (9.18, 3.77) and CO (6.53, 2.97), respectively. The associations were more pronounced with higher temperatures and lower relative humidity levels. We concluded that acute and subchronic exposure to higher atmospheric concentrations of all the pollutants studied were associated with greater severity of ARF. The greatest increases in LOS and risk of death occurred with hot and dry weather.

## Introduction

Urban air pollution is recognized as a major factor that harms human health due to the increase in morbidity and mortality from cardiovascular and respiratory diseases, cancer, diabetes and systemic implications and worsening of chronic diseases, in addition to compromising the integrity of the environment. The World Health Organization warns that more than 90% of the world’s population lives in places where air pollution rates are above the recommended limits. The main air pollutants are particulate matter (PM) and gaseous pollutants^1^. The effects on human health depend on external factors, such as the concentration of pollutants in the atmosphere, and on body characteristics, such as the defenses of an individual’s respiratory and immune systems. It is estimated that air pollutants contribute to approximately 7 million annual deaths worldwide, including 300,000 deaths among children under 5 years of age^2,3^, and a loss of life expectancy of approximately 3 years^4^. In Brazil, Cohen et al. reported more than 50,000 deaths secondary to air pollution in 2015, although other authors reported more than 100,000 deaths in the same period^5,6^.

Among children and adolescents, numerous associations have been reported between environmental concentrations of pollutants and asthma, wheezing, allergic rhinitis, pneumonia, and loss of lung function^7,8^. In 2015, it was estimated that 13% of new cases of asthma among children and adolescents were associated with exposure to NO_2_, with 150,000 cases occurring in Brazil and Paraguay^9^. In California (USA), a study that included more than 3600 children showed that exposure to particulate matter (PM) and NO increased the prevalence of respiratory symptoms and medication use among asthmatic children compared to children without asthma, emphasizing the susceptibility of patients with asthma. Systematic reviews also showed a 12 to 30% increase in the risk of pneumonia among children exposed to NO_2_ and PM^10,11^. Exposure during pregnancy is also related to impaired neonatal outcomes such as prematurity and low birth weight^12^.

Less studied is the influence of these exposures during the first two years of life. In this age group, the respiratory system is still developing, so diseases can have acute and long-term repercussions in adult life. Infants are often affected by respiratory infections, whose main etiological agents are respiratory viruses, and the effects of these infections and pollutants on the respiratory system may be potentiated ^13^.

In addition to acute exposure, another aspect that has been little studied is the possible effect of subchronic exposure to pollutants on respiratory infections in infants. Karr et al.^14^ showed that greater exposure to PM_2.5_ in the month prior to hospitalization was associated with a greater risk of hospitalization of infants due to bronchiolitis. The effects of pollutants, especially PM, on the modulation of the immune response to respiratory virus infections were shown in previous studies in animal models and in vitro in cells of the human respiratory system^15,16^.

Respiratory infections in infants are frequent in São Paulo, which has high levels of air pollution. Cases of severe respiratory infections that present as ARF are compulsorily reported and reached more than 1400 cases among infants in São Paulo in 2019^17^.

Knowing the influence of pollutants on the severity of ARF can contribute to health policies that prevent ARF and reduce morbidity and mortality among infants. In the present study, the authors analyzed the influence of acute and subchronic exposures to the main urban air pollutants on the severity of ARF in infants hospitalized in São Paulo.

## Materials and methods

We conducted a cross-sectional population-based study that included information on hospitalization of infants with ARF in São Paulo from 2010 to 2019.

The data were collected from the Influenza Epidemiological Surveillance Information System^17^. This information system is a platform for reporting cases of ARF among individuals with flu-like syndrome hospitalized in Brazil, according to clinical criteria. The system is supplied with data from public and private networks and covers several etiological agents. The reporting of hospitalized ARF is compulsory. The clinical criteria for infants are flu-like syndrome (sudden onset of fever and respiratory symptoms such as cough, runny nose and nasal obstruction in the absence of another specific diagnosis) and dyspnea/respiratory distress or O2 saturation lower than 95% on room air or cyanosis of the lips or face, flaring of the nose, intercostal retraction, dehydration and loss of appetite. The database reports the presence of preexisting pediatric morbidities. For blank responses, no comorbidities were assumed, and data were considered missing when reported as “unknown information”^17^.

The atmospheric concentrations of primary pollutants and meteorological data were collected from the computerized system of the Environmental Company of the State of São Paulo^18^. These data comprise daily values of the concentrations of pollutants expressed in µg/m^3^ for all pollutants, except for CO, whose measurements are expressed in parts per million (ppm). The daily averages for temperature (degrees Celsius) and relative humidity (RH) were considered. Concentration measurements of pollutants, temperature, and RH from 22 stations located in the city were considered. The correlation between the measurements from the stations was tested to calculate the correlation coefficient for all of them. No stations were found whose coefficients were less than 0.4 in relation to the others, which made us choose to consider all measurements.

Data were analyzed using STATA 16.0® software^19^. For acute exposure, the daily means of pollutant concentrations 1, 3 and 7 days before the date of hospitalization were considered. The associations related to increases of 10 µg/m^3^ in the average atmospheric concentrations of PM, SO_2_ and NO_2_ and of 0.86 ppm of CO were considered. For subchronic exposure, the mean concentrations of pollutants in the previous month and in the 60 days prior to hospitalization were considered, except for 10 days immediately prior to hospitalization. The outcomes considered for the evaluation of severity were hospitalization time and progression to death. Generalized linear models were used for the outcome “length of hospital stay” (LOS) to calculate the differences in days, according to exposure. The association between pollutant exposure and the dichotomous “death” outcome was used in logistic regression models. A cut-off point of 5% was established for the probability to be wrong when null hypothesis is rejected. All models were adjusted for temperature and RH. When there was interaction between the pollutant and these environmental conditions, the adjustments were maintained, but separate models were built for each temperature/humidity level, divided according to the median. This allowed for better interpretation of the nature of the interaction. Likewise, it was decided not to adjust for comorbidities but to present separate models. Thus, the effect size for patients with and without comorbidities could be observed.

## Results

The ARF database included 7364 cases in the study period. There was a predominance of male infants in the first year of life. Most hospitalizations occurred among infants without comorbidities and without a confirmed etiological diagnosis. Among the agents identified, influenza virus predominated. The demographic and clinical characteristics of the included infants are presented in Table 1.

**Table 1:** Demographic, clinical, and etiological characteristics of infants with acute respiratory failure.

Characteristics	
Age	7.7 months (3.0–13.1)*
Sex (M x F)	57% × 43%
Length of hospital stay	7 days (04–13)*
Mortality rate	2.6% (193/7364)
Comorbidities	N (%)
Absent	5817 (79.0)
Heart diseases	257 (3.5)
Chronic lung diseases	519 (7.1)
Others	1017 (13.8)
Etiologies	N (%)
Influenza Virus	824 (11.2)
Other respiratory viruses	1309 (17.8)
Other pathogens	714 (9.7)
Not specified	4515 (61.3)

*Median (25th–75th percentile); *M* = male, *F* = female.

**Figure 1:**
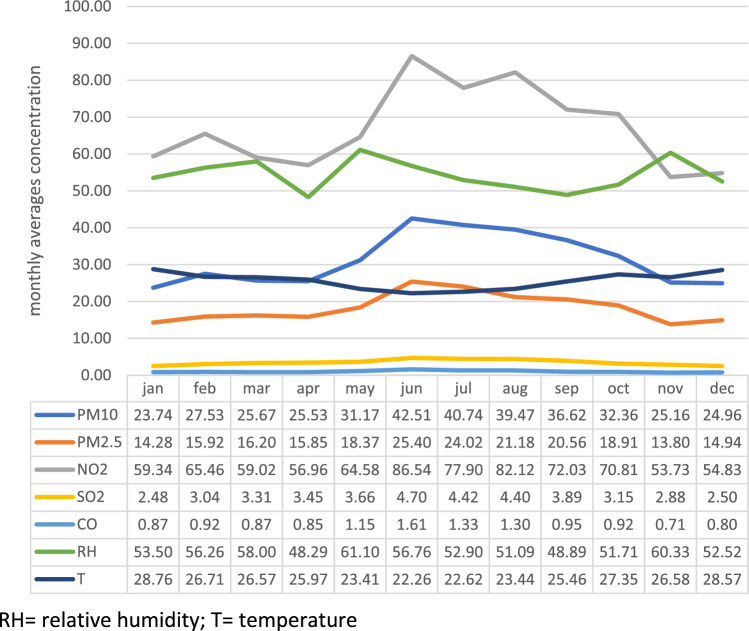
Graphic 1: Distribution of the mean concentrations of pollutants (PM_10_, PM_2.5_, NO_2_, SO_2_ in µg/m^3^ and CO in ppm), temperature (degrees Celsius) and relative humidity (percentage), according to month, from 2010 to 2019, in São Paulo. *RH* relative humidity; *T* = temperature.

The highest concentrations of atmospheric pollutants occurred between May and November, when the lowest RH also occurred. (Fig. 1).

Acute exposure to higher concentrations of all pollutants was associated with greater severity. Higher NO_2_ concentrations were associated with increased LOS and increased risk of death, without interactions with temperature or RH. Higher exposure to SO_2_ was associated with increased LOS, and when there was an interaction with temperature, there were associations with higher temperatures. SO_2_ was also positively associated with the risk of death; when there were interactions, there were associations with higher temperatures and lower RH. Higher exposure to CO was associated with increased LOS and risk of death, and when there were interactions, there were associations with higher RH. Higher concentrations of PM_10_ and PM_2.5_ 3 days before admission were associated with increased LOS, without interactions with temperature or RH. (Table 2).

**Table 2:** Associations between daily mean concentration of pollutants 1, 3 and 7 days before admission and severity outcomes in infants with acute respiratory failure.

Length of stay													
Pollutants		1 day before admission				3 days before admission				7 days before admission			
		Coef	P	CI		Coef	P	CI		Coef	P	CI	
MP_10_													
No interactions		–	–	–		**0.30**	**0.01**	**0.06**	**0.54**	–	–	–	
Interaction with temperature	lo	0.29	0.11	− 0.06	0.64	–	–	–		0.29	0.12	− 0.08	0.67
	hi	0.32	0.07	− 0.03	0.67	–	–	–		0.19	0.283	− 0.16	0.54
MP_2.5_													
No interactions		–	–	–		**0.37**	**0.05**	**0.00**	**0.74**	0.23	0.25	− 0.15	0.60
Interaction with temperature	lo	0.18	0.50	− 0.34	0.70	–	–	–		–	–	–	
	hi	0.34	0.23	− 0.21	0.89	–	–	–		–	–	–	
NO_2_													
No interactions		**0.24**	**<0.01 **	**0.10**	**0.38**	0.13	0.06	− 0.01	0.27	**0.16**	**0.03**	**0.02**	**0.30**
SO_2_													
No interactions		**6.64**	**<0.01**	**4.64**	**8.63**	–	–	–		**5.07**	**<0.01 **	**3.10**	**7.05**
Interaction with temperature	lo	–	–	–		1.70	0.20	− 0.90	4.30	–	–	–	
	hi	–	–	–		**10.81**	**<0.01**	**7.45**	**14.17**	–	–	–	
CO													
No interactions		–	–	–		**0.71**	**0.01**	**0.23**	**1.19**	**0.87**	**<0.01 **	**0.37**	**1.37**
Interaction with relative humidity	lo	0.42	0.18	− 0.20	1.03	–	–	–		–	–	–	
	**hi**	**1.82**	**<0.01 **	0**.91**	**2.72**	–	–	–		–	–	–	

Chance of death													
Pollutants		1 day before admission				3 days before admission				7 days before admission			
		OR	P	CI		OR	P	CI		OR	P	CI	
MP_10_													
No interactions		1.07	0.07	0.99	1.16	–	–	–		0.98	0.74	0.86	1.11
Interaction with temperature	lo	–	–	–		1.07	0.29	0.95	1.20	–	–	–	
	hi	–	–	–		0.99	0.85	0.89	1.10	–	–	–	
MP_2.5_													
No interactions		1.08	0.24	0.95	1.23	–	–	–		1.04	0.69	0.86	1.27
Interaction with temperature	lo	–	–	–		1.12	0.25	0.93	1.35	–	–	–	
	hi	–	–	–		1.00	0.99	0.83	1.21	–	–	–	
NO_2_													
No interactions		**1.06**	**<0.01**	**1.02**	**1.12**	**1.05**	**0.04**	**1.00**	**1.09**	0.97	0.71	0.92	1.06
SO_2_													
No interactions		–	–	–		**2.04**	**<0.01 **	**1.33**	**3.13**	0.553	0.17	0.24	1.30
Interaction with temperature	lo	1.21	0.55	0.64	2.30	–	–	–		–	–	–	
	hi	**3.64**	**<0.01**	**2.05**	**6.48**	–	–	–		–	–	–	
Interaction with relative humidity	lo	**2.46**	**<0.01 **	** 1.47 **	**4.13**	–	–	–		–	–	–	
	hi	1.97	0.06	0.97	4.02	–	–	–		–	–	–	
CO													
No interactions		–	–	–		**1.19**	**0.01**	**1.04**	**1.37**	0.94	0.61	0.73	1.20
Interaction with temperature	lo	1.03	0.79	0.84	1.27	–	–	–		–	–	–	
	hi	**1.28**	**0.02**	**1.04**	**1.57**	–	–	–		–	–	–	

Coef = Increase in length of hospitalization for increases of 10 mcg/m^3^ in MP_10_, MP_2.5_, NO2, SO_2_ and of 0.86 ppm in CO; OR = Increase in chance of death for increases of 10 mcg/ m^3^ in MP_10_, MP_2.5_, NO_2_, SO_2_ and of 0.86 ppm in CO. The models are presented as single models when there was no interaction between the pollutant and temperature/relative humidity (No interactions). When there was interaction, the models were presented separately to observe the difference in effect with low/hi temperature and low/hi relative humidity. lo = below the median; hi = above the median. *p* = *p* value; CI = 95% confidence interval.

Significant values are in Bold.

Acute exposure to higher concentrations of all pollutants studied was associated with greater severity in children without comorbidities. Higher concentrations of PM_10_, SO2 and CO were also associated with increased LOS among children with comorbidities. Higher concentrations of SO_2_ and CO were associated with longer hospital stays both among children without comorbidities and among those with comorbidities, and the increase in LOS was more pronounced among children with comorbidities. The highest concentrations of NO_2_, SO_2_ and CO were associated with an increased risk of death among previously healthy children. (Table 3).

**Table 3:** Associations between daily concentration of pollutants 1, 3 and 7 days before admission and severity outcomes in infants with acute respiratory failure with and without comorbidities.

Length of stay												
CM (−) n= 5030 CM(+) n=1323												
Pollutants	1 day before admission				3 days before admission				7 days before admission			
	Coef	P	CI		Coef	P	CI		Coef	P	CI	
MP_10_												
CM (−)	**0.32**	**0.01**	**0.08**	**0.57**	0.12	0.36	− 0.13	0.36	**0.26**	**0.05**	**0.00**	**0.51**
CM (+)	0.34	0.29	− 0.29	0.96	**0.76**	**0.02**	**0.15**	**1.38**	0.204	0.545	− 0.46	0.86
MP_2.5_												
CM (−)	**0.39**	**0.04**	**0.01**	**0.78**	0.16	0.41	− 0.22	0.55	0.34	0.09	− 0.05	0.73
CM (+)	− 0.11	0.83	− 1.06	0.85	0.79	0.10	− 0.16	1.74	− 0.37	0.47	− 1.36	0.63
NO_2_												
CM (−)	**0.20**	**0.01**	**0.06**	**0.35**	0.10	0.18	− 0.05	0.24	0.13	0.08	− 0.01	0.28
CM (+)	0.25	0.18	− 0.11	0.62	0.17	0.37	− 0.19	0.52	0.19	0.32	− 0.18	0.55
SO_2_												
CM (−)	**4.52**	**<0.01**	**2.43**	**6.60**	**3.62**	**<0.01**	**1.54**	**5.70**	**3.77**	**<0.01**	**1.68**	**5.86**
CM (+)	**10.87**	**<0.01**	**5.76**	**15.99**	**8.72**	**<0.01**	**3.62**	**13.82**	**6.36**	**0.01**	**1.41**	**11.30**
CO												
CM (−)	**0.66**	**0.02**	**0.12**	**1.15**	0.35	0.17	− 0.15	0.86	**0.59**	**0.03**	**0.06**	**1.11**
CM (+)	0.85	0.20	− 0.42	2.06	**1.52**	**0.01**	**0.31**	**2.72**	**1.42**	**0.03**	**0.14**	**2.70**

Chance of death												
CM(–) n= 6038 CM(+) n= 1067												
	1 day before admission				3 days before admission				7 days before admission			
	OR	P	CI		OR	P	CI		OR	P	CI	
MP_10_												
CM (−)	1.09	0.06	1.00	1.19	1.06	0.23	0.97	1.16	0.91	0.26	0.76	1.08
CM (+)	1.00	0.94	0.86	1.15	1.01	0.85	0.88	1.16	1.07	0.5	0.88	1.30
MP_2.5_												
CM (−)	1.01	0.24	0.94	1.28	1.07	0.38	0.95	1.26	0.92	0.54	0.70	1.20
CM (+)	0.98	0.86	0.78	1.23	1.09	0.44	0.88	1.36	1.17	0.29	0.88	1.56
NO_2_												
CM (−)	**1.06**	**0.02**	**1.01**	**1.11**	1.05	0.1	0.10	1.10	0.95	0.26	0.86	1.04
CM (+)	1.05	0.21	0.97	1.14	1.04	0.3	0.96	1.13	1.03	0.6	0.93	1.14
SO_2_												
CM (−)	**2.81**	**<0.01 **	**1.74**	**4.53**	**2.49**	**<0.01**	**1.52**	**4.07**	0.33	0.08	0.10	1.12
CM (+)	1.21	0.65	0.53	2.79	1.05	0.93	0.44	2.45	0.73	0.62	0.22	2.50
CO												
CM (−)	**1.24**	**0.01**	**1.05 **	**1.46**	1.18	0.06	0.99	1.40	0.87	0.41	0.62	1.22
CM (+)	1.08	0.58	0.83	1.39	1.18	0.17	0.93	1.50	0.98	0.93	0.67	1.43

Coef = Increase in length of hospitalization for increases of 10 mcg/m^3^ in MP_10_, MP_2.5_, NO2, SO_2_ and of 0.86 ppm in CO; OR = Increase in chance of death for increases of 10 mcg/ m^3^ in MP_10_, MP_2.5_, NO_2_, SO_2_ and of 0.86 ppm in CO; CM(−) = absence of comorbidities; CM(+) = presence of comorbidities; *p* = *p* value; CI = 95% confidence interval.

Significant values are in Bold.

Higher mean SO_2_ and CO concentrations in the month prior to admission were associated with higher LOS when temperatures were higher. There were no statistically significant associations for the other studied pollutants and LOS or for the risk of death. Higher SO_2_ concentrations in the last 60 days before admission were associated with increases in LOS, and this occurred at both low and high temperatures and RH. Higher CO concentrations were associated with increases in LOS with lower temperatures and higher RH. There were no significant associations with the risk of death (Table 4).

**Table 4:** Associations between subchronic exposures (mean of 30 and 60 days) and severity outcomes in infants with acute respiratory failure.

Lenght of stay									
Pollutants		Mean 30 days before admission				Mean 60 days before admission			
		Coef	P	CI		Coef	P	CI	
MP_10_									
No interactions		0.04	0.66	− 0.15	0.25	0.01	0.89	− 0.18	0.21
MP_2.5_									
No interactions		− 0.03	0.87	− 0.34	0.29	− 0.07	0.66	− 0.38	0.24
NO_2_									
No interactions		0.03	0.69	− 0.10	0.15	0.10	0.88	− 0.11	0.13
SO_2_									
Interaction with temperature	Lo	**2.77**	**0.02**	**0.49**	**5.06**	**3.69**	**0.01**	1.14	6.23
	Hi	**9.18**	**<0.01**	**5.35**	**13.06**	**3.77**	**0.02**	0.66	6.87
Interaction with relative humidity	Lo	–	–	–		**3.59**	**0.01**	0.91	6.27
	Hi	–	–	–		**5.41**	**<0.01**	2.15	8.66
CO									
Interaction with temperature	Lo	1.1	0.09	− 0.16	2.36	**2.44**	**0.01**	**0.84**	**4.03**
	Hi	**6.53**	**<0.01**	**3.67**	**9.39**	1.56	0.18	− 0.70	3.83
Interaction with	Lo	–	–	–		1.21	0.22	− 0.71	3.14
relative humidity	Hi	–	–	–		**2.97**	**<0.01**	**1.22**	**4.72**

Chance of death									
Pollutants	Mean 30 days before admission				Mean 60 days before admission				
	OR	P	CI		OR	P	CI		
MP_10_									
No interactions	0.96	0.46	0.87	1.07	0.97	0.52	0.88	1.07	
MP_2.5_									
No interactions	1.02	0.85	0.86	1.20	1.02	0.79	0.87	1.20	
NO_2_									
No interactions	0.98	0.6	0.93	1.05	0.99	0.68	0.93	1.05	
SO_2_									
No interactions	0.51	0.11	0.22	1.17	0.58	0.19	0.25	1.32	
CO									
No interactions	0.68	0.13	0.41	1.12	0.59	0.08	0.32	1.07	

Coef = Increase in lenght of hospitalization for increases of 10 mcg/m^3^ in mean of MP_10_, MP_2.5_, NO_2_, SO_2_ and of 0.86 ppm in mean of CO over the period**;** OR = Increase in chance of death for increases of 10 mcg/ m^3^ in mean of MP_10_, MP_2.5_, NO_2_, SO_2_ and of 0.86 ppm in mean of CO over the period; Lo = below the median; Hi = above the median**;** *p* = *p* value; CI = 95% confidence interval.

Significant values are in Bold.

The highest mean SO2 concentrations in the last 30 days increased the risk of death among children with comorbidities, and the highest CO concentrations in the last 60 days were associated with greater LOS among children with comorbidities. Among previously healthy children, higher SO2 averages in the last 30 days and 60 days before admission were associated with an increase in LOS, and increases in CO in the last 60 days were associated with a greater risk of death among previously healthy children (Table 5).

**Table 5:** Associations between subchronic exposures (mean of 30 and 60 days) and severity outcomes in infants with acute respiratory failure with or without comorbidities.

Length of stay								
CM (−) n = 5030 CM (+) n = 1323								
Pollutants	Mean 30 days before admission				Mean 60 days before admission			
	Coef	P	CI		Coef	P	CI	
MP_10_								
CM (−)	0.04	0.69	− 0.16	0.25	0.03	0.80	− 0.18	0.23
CM (+)	0.02	0.93	− 0.50	0.55	− 0.05	0.85	− 0.56	0.46
MP_2.5_								
CM (−)	0.04	0.79	− 0.28	0.37	0.02	0.90	− 0.30	0.34
CM (+)	− 0.35	0.41	− 1.16	0.47	− 0.44	0.29	− 1.24	0.37
NO_2_								
CM (−)	0.03	0.62	− 0.09	0.16	0.02	0.73	− 0.10	0.15
CM (+)	− 0.02	0.89	− 0.33	0.29	− 0.05	0.74	− 0.36	0.25
SO_2_								
CM (−)	**3.46**	**<0.01**	**1.46**	**5.45**	**3.51**	**<0.01**	**1.50**	**5.52**
CM (+)	2.95	0.23	− 1.90	7.80	2.61	0.29	− 2.25	7.46
CO								
CM (−)	1.03	0.08	− 0.13	2.18	0.96	0.17	− 0.40	2.32
CM (+)	1.90	0.18	− 0.86	4.67	**4.22**	**0.01**	**0.91**	**7.52**

Chance of death								
CM (−) n=5805 CM (+) n=1538								
Pollutants	Mean 30 days before admission				Mean 60 days before admission			
	OR	P	CI		OR	P	CI	
MP_10_								
CM (−)	0.98	0.77	0.85	1.12	0.99	0.86	0.86	1.13
CM (+)	0.93	0.37	0.80	1.09	0.94	0.4	0.80	1.09
MP_2.5_								
CM(−)	1.06	0.61	0.85	1.32	1.07	0.54	0.86	1.33
CM(+)	0.94	0.64	0.74	1.21	0.95	0.68	0.75	1.21
NO_2_								
CM (−)	1.00	0.99	0.92	1.09	1.01	0.9	0.93	1.09
CM (+)	0.96	0.34	0.87	1.05	0.96	0.38	0.88	1.05
SO_2_								
CM (−)	0.67	0.47	0.23	1.98	0.77	0.64	0.26	2.26
CM (+)	**0.27**	**0.05**	**0.07**	**1.01**	0.30	0.07	0.08	1.13
CO								
CM (−)	0.54	0.09	0.26	1.10	**0.38**	**0.03**	**0.16**	**0.92**
CM (+)	0.73	0.39	0.35	1.51	0.71	0.44	0.30	1.70

Coef = Increase in length of hospitalization for increases of 10 mcg/m^3^ in mean of MP_10_, MP_2.5_, NO_2_, SO_2_ and of 0.86 ppm in mean of CO over the period**;** OR = Increase in chance of death for increases of 10 mcg/ m^3^ in mean of MP_10_, MP_2.5_, NO_2_, SO_2_ and of 0.86 ppm in mean of CO over the period;CM(−) = absence of comorbidities; CM(+) = presence of comorbidities; *p* = *p* value; CI = 95% confidence interval.

Significant values are in Bold.

## Discussion

In this population-based study, exposure to higher concentrations of air pollutants was associated with a greater severity of ARF in hospitalized infants. During the week before admission, higher atmospheric levels of PM and gaseous pollutants increased the length of hospitalization and the odds of death. Subchronic exposure was also associated with a longer hospital stay.

The study was conducted in São Paulo city, where the main emitting sources of atmospheric pollutants are vehicles and industry. In 2019, the resident population was estimated at 21.9 million, and the vehicle fleet was estimated at 7,324,690^20,21^. As expected, the highest concentrations of air pollutants occurred in the months with drier climates, when the dispersion of pollutants was more compromised.

Although previous studies have indicated that exposure to air pollutants is a serious risk factor for respiratory diseases in adults, children, and adolescents, few studies have focused on respiratory health in the first two years of life. Most studies that included children were conducted among preschoolers and schoolchildren, who usually spend much of their time in open environments. A 2018 systematic review that studied the effects of outdoor pollution on bronchiolitis included only 8 epidemiological studies^22^. However, infants, especially those living in countries with milder climates, low household incomes, and homes without insulation, may also be at risk from outdoor pollutants.

The first years of life are a period of susceptibility to ARF due to the anatomical and functional immaturity of infants’ respiratory and immune systems, in addition to the higher tidal volume-to-body weight ratio^13^. The sample analyzed in the present study represented cases of severe respiratory failure caused by the most frequent infectious agents in this age group.

The greater severity in infants exposed to higher concentrations of air pollutants in the days before hospitalization is in agreement with the findings of previous studies that addressed bronchiolitis. Nenna et al.^23^ showed a positive association between the incidence of respiratory syncytial virus (RSV) infections and atmospheric concentrations of benzene, NOx, SO2 and PM, as did an epidemiological study of RSV in infants exposed to higher concentrations of PM_10_^24^. The influence of air pollutants has also been reported for SARS-CoV-2 and influenza^25,26^.

Our results highlighted an increase of approximately 130% in the risk of death with greater acute exposure to SO_2_, more than 20% for CO and between 5 and 8% for NO_2_ and PM. Additionally, an increase in LOS of more than 5 days associated with acute exposure to higher SO_2_ concentrations was noteworthy. Even an increase of slightly less than 1 day for all other pollutants has population repercussions, such as increased costs for the health system. These estimates represent an enormous impact, considering the large number of individuals who suffer these exposures.

There are several possible mechanisms that explain the effects of pollutants on the severity of ARF. PM affects the airways through inflammatory mechanisms and cytotoxicity. Changes in the immune response have also been described^27^. The effects of PM on the human body are diverse, as PM consists of a mixture of particles that, depending on their size, reach different regions of the respiratory system. Despite being usually classified according to aerodynamic diameter, PM is also varied in its constitution depending mainly on the emitting sources. In vivo studies have contributed to elucidating the etiopathogenic mechanisms involved. When RSV associated with PM_10_ comes into contact with respiratory epithelial cells, there is a greater increase in the secretion of inflammatory mediators (IL-6 and IL-8) compared to infection without PM_10_^28^. Other studies report that PM_2.5_ can induce epigenetic changes through microRNA overexpression associated with inflammation and susceptibility to infections, in addition to activating the recruitment of inflammatory cells, the secretion of proinflammatory factors, the production of free radicals and the destruction of the extracellular matrix^29,30^.

Prolonged exposure to high concentrations of pollutants is also associated with longer hospital stays. Subchronic exposures have rarely been explored in previous studies that focused on acute exposures and chronic exposures (6 months to lifetime). Prolonged exposure to PM can alter the immune response and increase susceptibility to influenza virus infections in children^31,32^. Interestingly, in animal studies, acute exposure to PM_2.5_ increased the survival of mice infected with influenza compared to those not exposed to PM; however, prolonged exposure led to exhaustion of the animals’ immune system, favoring the progression of infection^32^. Previous studies have indicated that exposure to ultrafine particles of PM induces inflammation and allergic responses and compromises the production of interferon gamma necessary for defense against infections^15^. There is also similar evidence regarding gaseous pollutants. In vitro studies have shown the immunological modulation of NOx with the production of inflammatory cytokines, increased expression of viral receptors and favoring of viral replication and action^33^. The effect of subchronic exposure on bronchiolitis in infants was analyzed in a case‒control study in California. In agreement with our results, exposure to PM_2.5_ in the previous month was associated with a higher risk of hospitalization^14^. In vivo studies have also shown that PM_10_ can remain in the respiratory system for long periods before being eliminated. This prolonged exposure of epithelial cells may contribute to the secretion of proinflammatory cytokines such as IL-1 beta in response to infection by the influenza virus through an NLRP3-dependent mechanism^8,31^.

In the present study, the gaseous pollutant most strongly associated with the severity of ARF was SO_2_. Sulfur oxides react with atmospheric compounds, forming small particles that penetrate deeply into the lungs and can cause or aggravate respiratory diseases. Respiratory symptoms can occur as rapidly as 10 min, and SO_2_ exposure is associated with increased visits to emergency departments^34^. In agreement with these data, our results showed an important association of higher acute exposure to SO_2_. The increase in hospitalization time reached 6.6 days, and there was a 360% risk of death with high temperature. The results also suggested an important subchronic effect on the LOS, with an increase of 9.2 days for higher exposures during the 30 days prior to hospitalization. The comparison of groups suggested that the effect of chronic exposure occurred in previously healthy infants and is even more relevant in those with comorbidities.

The effect of acute and subchronic exposure to CO on the severity of ARF, as in the present study, has also been less studied^35^. It is known that CO forms a stable bond with hemoglobin, preventing oxygen transport and causing tissue hypoxia. Additionally, mechanisms of cellular toxicity, such as oxidative stress, apoptosis, inflammation, and lesions associated with the immune response, have been reported^36^.

NO_2_ elevations were also associated with severity outcomes. In addition to increases in atmospheric NO_2_ being associated with a 5 to 6% increase in the risk of death for acute exposure, they also corresponded to increased LOS for acute and subchronic exposure. No previous studies were found in the literature on the influence of subchronic exposure to NO_2_ on the respiratory health of infants. Importantly, NO_2_ is a precursor of a range of secondary pollutants. Through a sequence of photochemical reactions initiated by solar radiation, NO_2_ produces oxidants that are converted into ammonium salts, forming organic particles, nitrates and sulfates that compose the PM^1^. Thus, the associations found in the present study may partially reflect the influence of PM on the severity of ARF outcomes.

The greater severity of exposure to pollutants on days with dry weather was noteworthy, as the dispersion of atmospheric pollutants is smaller under these conditions^17^. In the present study, the influence of temperature was more marked in both acute and subchronic exposures, with the greatest severity of ARF being observed in high exposures to SO2 and CO in hot weather. The influence of RH was not as relevant since there were positive associations with severity in low- and high-RH situations. The study design did not allow for a more in-depth analysis of the influence of temperature since other factors, such as rainfall and wind index, also interfere^17^.

The smaller number of individuals with comorbidities may have impaired the comparisons between infants with and without comorbidities. Nevertheless, in situations in which statistically significant associations were observed in both groups, greater acute exposure to SO_2_ was associated with a greater increase in hospitalization time among those with comorbidities. Notably, higher acute exposure to SO_2_ was associated with an increase of more than 10 days of hospitalization among children with comorbidities, and subchronic exposure was associated with an almost 30% increase in their risk of death. According to previous studies, the presence of chronic diseases increases susceptibility to the deleterious effects of air pollutants in adults^1^. There are fewer studies on the effects of air pollution on children with comorbidities. Rosenquist et al.^37^ suggested that children with allergic asthma are more susceptible to the effects of PM_2.5_ than children with nonallergic asthma. Other studies have also reported the greater vulnerability of children with rheumatological diseases^38^. On the other hand, our results draw attention to the serious effects of air pollution on the health of previously healthy children.

The sample size and the design of this study did not allow a comparative analysis between the different etiologies of ARF. Future studies may propose the same associations considering the etiologies. Due to the nature of the database, some clinical information was missing. However, as this lack of information was not correlated with exposure, it is unlikely that there was a bias toward one side or the other, affecting at most the size of the estimate.

## Conclusions

The results contribute to the characterization of the possible effects of urban air pollution on the health of infants. Higher atmospheric concentrations of all pollutants studied were associated with greater severity of ARF, especially during periods of hot and dry weather.

## Data Availability

The data supporting reported results can be found at: https://opendatasus.saude.gov.br/dataset/srag-2009-2012—For each year (2009–2010-2011 and 2012)—Explorar – baixar. https://opendatasus.saude.gov.br/dataset/srag-2013-2018—For each year (2013–2014–2015–2016–2017 and 2019)—Explorar–baixar. https://opendatasus.saude.gov.br/dataset/srag-2019—Explorar—baixar. link: https://docs.google.com/spreadsheets/d/1dbundy5nhSmXJJC6YYtm9IytajKrQyaJ/edit?usp=sharing&ouid=103230360813112929171&rtpof=true&sd=true
